# Supplementary material for: Indirect chiral magnetic exchange through Dzyaloshinskii–Moriya-enhanced RKKY interactions in manganese oxide chains on Ir(100)
Source: Nat Commun. 2019 Jun 13;10:2610. doi: 10.1038/s41467-019-10515-3 (PMC6565667; doi:10.1038/s41467-019-10515-3)
Supplement: Supplementary file 1 — Supplementary Information [file 41467_2019_10515_MOESM1_ESM.pdf]

Supplementary Information for: Schmitt *et al.* "Indirect Chiral Magnetic Exchange through Dzyaloshinskii-Moriya–Enhanced RKKY Interactions in Manganese Oxide Chains on Ir(100)

Martin Schmitt,[1,*] Paolo Moras,[2] Gustav Bihlmayer,[3] Ryan Cotsakis,[1,4] Matthias Vogt,[1] Jeannette Kemmer,[1] Abderrezak Belabbes,[5] Polina M. Sheverdyaeva,[2] Asish K. Kundu,[6] Carlo Carbone,[2] Stefan Blügel,[3] and Matthias Bode[1,7]

[1]*Physikalisches Institut, Experimentelle Physik II, Universität Würzburg, Am Hubland, 97074 Würzburg, Germany*

[2]*Istituto di Struttura della Materia-CNR (ISM-CNR), Trieste, Italy*

[3]*Peter Grünberg Institut and Institute for Advanced Simulation, Forschungszentrum Jülich & JARA, 52425 Jülich, Germany*

[4]*University of British Columbia, 2329 West Mall, Vancouver, BC Canada*

[5]*Physical Science and Engineering Division, King Abdullah University of Science & Technology (KAUST), Thuwal 23955-6900, Saudi Arabia*

[6]*International Center for Theoretical Physics (ICTP), Trieste, Italy*

[7]*Wilhelm Conrad Röntgen-Center for Complex Material Systems (RCCM), Universität Würzburg, Am Hubland, 97074 Würzburg, Germany*

(Dated: May 4, 2019)

Supplementary Note 1: Temperature-dependent ARPES data

The photoemission spectra of MnO_2 chains on Ir(001) exhibit relatively broad peaks at any given k-value (Fig. 1e). Since the Ir-related bands in the very same spectra are well resolved, this cannot be an effect of insufficient statistics or resolution, but must be related to intrinsic factors, such as the overlap of many Mn-related bands, whose spectral weight changes as a function of k, and their hybridization with the substrate bands, as expected from the DFT calculations (Fig. 1f). In spite of this complexity, the Mn 3d features marked with symbols in Fig. 1e show a dispersion compatible with the 2× AFM ordering along the chains. The link between the electronic structure and the AFM ordering is reinforced in Supplementary Figure 1, which compares ARPES data taken at different temperatures. The electronic states of the RT data [Supplementary Figure 1(a)] are similar to those seen in the LT data [Supplementary Figure 1(b)], but much more broadened. This is not only an effect of phonon broadening, since the Ir-related states look very similar at the two temperatures. We interpret our observation as due to magnetic disorder at higher temperature, possibly coexisting with AFM short range correlations.

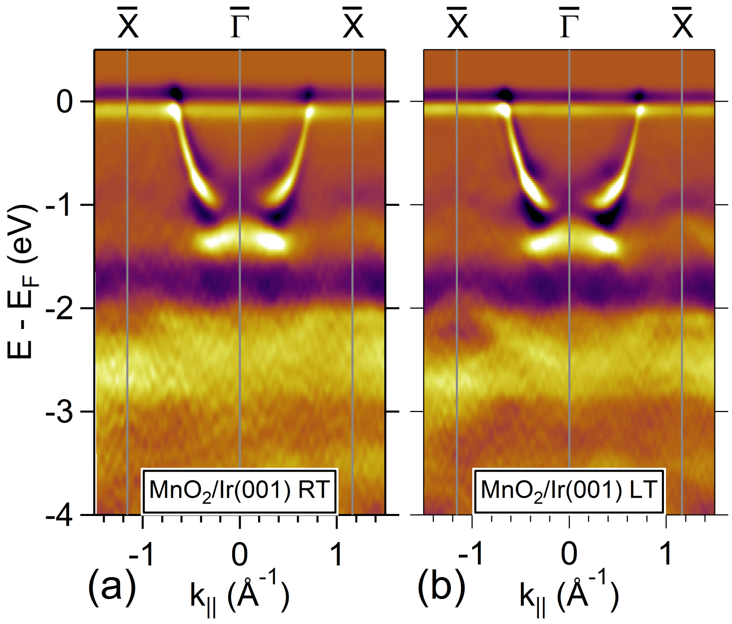

Supplementary Figure 1. Second derivative ARPES spectra of MnO_2 chains on Ir(001) measured at (a) RT and (b) LT with $h\nu = 150\,\text{eV}$.

Supplementary Note 2: Orbital symmetry of Mn-related states

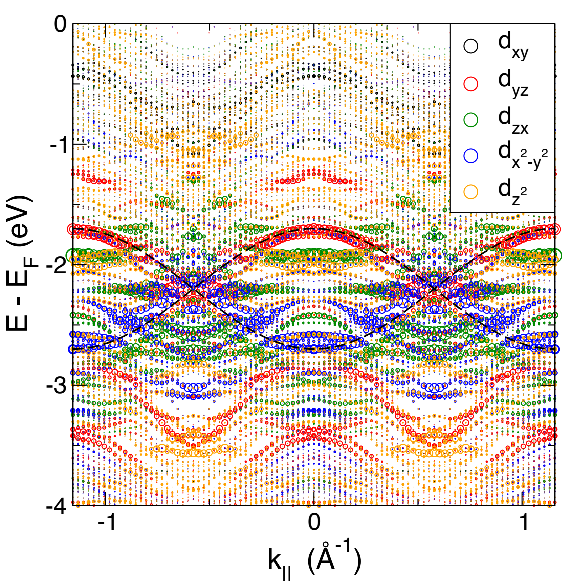

Supplementary Figure 2. Orbital decomposition of the band structure shown in Fig. 1f. Size and color of the circles indicate the specific Mn *d*-character as shown in the legend. It is clearly seen that the dispersive states are of d_{yz} and $d_{x^2-y^2}$ character (here, the Mn chains run in y-direction) while non-dispersive states between -2 and -3 eV are mainly of d_{zx} and d_{z^2} character. The dispersion of the d_{yz} and $d_{x^2-y^2}$ states is marked as in Fig. 1f. The calculation was performed for a (3×2) unit cell with an antiferromagnetic order of the Mn atoms and with the inclusion of SOC effects.

Supplementary Note 3: Identification of the MnO_2 easy magnetization direction

(a) SP-STM with out-of-plane magnetized tips

In order to determine the easy magnetization axes of the chiral (9×2) spin structure of MnO_2/Ir(001) we have performed experiments on Fe double-layer (DL) films epitaxially grown on W(110), a sample system that is particularly suited as a magnetic reference system. It is well-known from earlier studies [1–9] that the Fe DL on W(110) is ferromagnetic at low temperature and exhibits an out-of-plane easy axis. However, depending on the terrace width of the W(110) substrate two characteristic magnetization patterns are formed.

(i) At high miscut from the ideal (110) orientation the terraces are relatively narrow (typically 5 to 50 nm). As shown in Supplementary Figure 3(a) for a total coverage of about 1.3 atomic layers (AL) this results in Fe DL stripes which are attached to step edges and perpendicularly magnetized. Under these conditions adjacent Fe DL stripes couple antiparrallel due to the stray field-mediated dipolar interaction [1, 2, 5]. As represented by their dark and bright appearance in the differential conductance dI/dU map shown in Supplementary Figure 3(b), this magnetization pattern is clearly observed with a Cr-coated probe tip, thereby confirming its out-of-plane sensitivity.

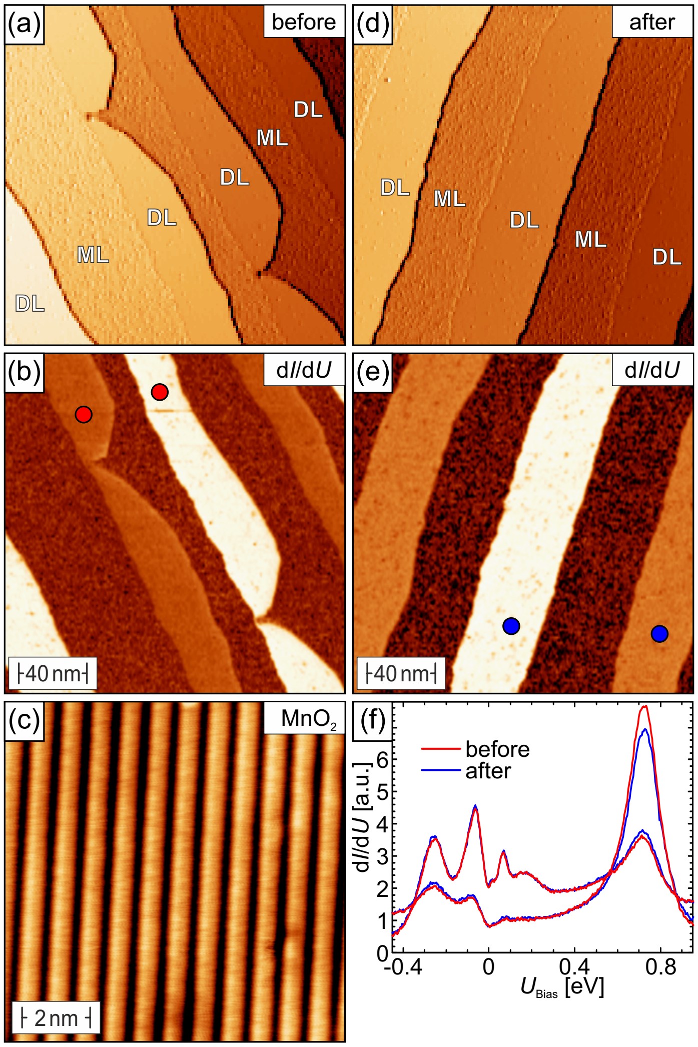

Supplementary Figure 3. (a) Topography and (b) the dI/dU signal of Fe double-layer (DL) stripes on W(110) measured with a Cr-coated tip, showing strong out-of-plane contrast. (c) SP-STM image of MnO_2 chains on Ir(001) taken with the same tip characterized in (b). No magnetic corrugation is resolved ($\Delta z_{SP} \lesssim 1$ pm). (d),(e) Control experiment performed on Fe DL stripes on W(110) after (c), confirming the strong out-of-plane contrast. (f) Tunneling spectra measured at the points indicated in (b) and (e). The spectra are virtually identical, verifying the absence of any unwanted tip change. Stabilization parameters: $U = 700$ mV, $I = 1.5$ nA, $U_{mod} = 10$ mV.

This out-of-plane sensitive tip has been used for SP-STM measurements on MnO_2/Ir(001). These data are presented in Supplementary Figure 3(c). However, within the noise level of our experimental setup of about 1 pm no magnetic corrugation Δz_{SP} could be observed, indicating that the magnetic moments in the MnO_2 chains are predominantly oriented within the surface plane, although quantification is difficult. To estimate an upper limit of the in-plane component we have to consider that the spin-polarized contrast in SP-STM scales with the cosine of the angle between tip and sample magnetization. Therefore, a relatively small out-of-plane rotation of only 30° with respect to the surface plane would already result in 50% of the maximal achievable magnetic contrast. Since the upper limit of Δz_{SP} observed in Supplementary Figure 3(c) with a verified out-of-plane tip is very low ($\lesssim 1$ pm) we are convinced that we can exclude such a large perpendicular component of the sample magnetization.

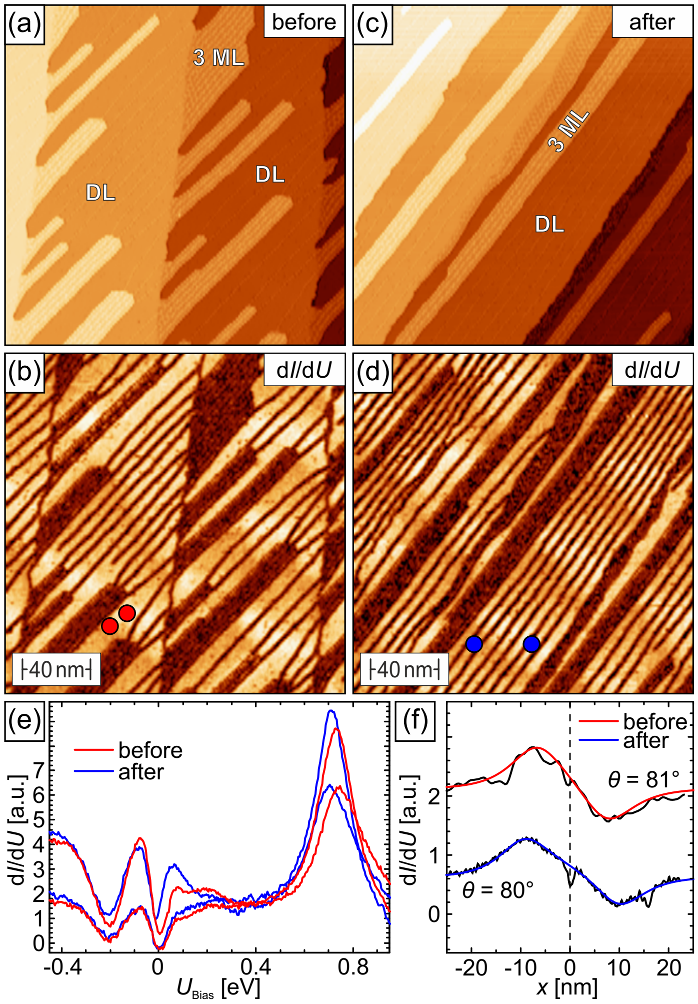

Supplementary Figure 4. Topography and differential conductance $\mathrm{d}I/\mathrm{d}U$ scans performed in Fe DL regions (a),(b) before and (c),(d) after intermittent measurements on MnO_2/Ir(001), respectively. In both cases the Fe-coated tip reveals a strong in-plane contrast. (e) Tunneling spectra measured at the points indicated in (b) and (d). The spectra are virtually identical, verifying the absence of any unwanted tip change. (f) Line sections drawn across domain walls in (b) and (d). The fit confirms that the tip predominantly oriented within the film plane. Stabilization parameters: $U = 700\,\mathrm{mV}$, $I = 1.5\,\mathrm{nA}$, $U_{\mathrm{mod}} = 10\,\mathrm{mV}$.

To exclude any unwanted tip changes when scanning MnO_2/Ir(001) we subsequently characterized the same tip again on the Fe DL on W(110). These data, which are presented in Supplementary Figure 3(d,e), confirm the out-of-plane sensitivity of this magnetically coated STM tip. The conclusion that the tip didn't change when scanning MnO_2/Ir(001) is also supported by the STS data presented in Supplementary Figure 3(f). Before and after the MnO_2/Ir(001) experiments we have taken tunneling spectra on each type of domains, up and down, of the Fe DL on W(110). The exact locations where the spectra were recorded are indicated by red and blue dots in Supplementary Figure 3(b) and (e), respectively. It is obvious that the spectra taken before (red lines) and after (blue) the MnO_2/Ir(001) experiments almost perfectly agree. Given the fact that even subtle tip changes often dramatically change the spectral intensity, presumably due to the strong dependence of the tunneling matrix element on the orbital character of the tip's electronic states, we believe that the tip remained completely unchanged during the course of our experiments.

(b) SP-STM with in-plane magnetized tips

(ii) At low miscut the terraces of the W(110) substrate are much wider (> 50 nm). As shown in various publications [3, 4, 6–9] this results in a magnetization pattern which is characterized by periodic reversals between up and down within every individual nanowire. The magnetic periodicity amounts to about 50 nm. The topography and magnetic dI/dU signal of such a sample surface as measured with the same Fe-coated tip *prior to* the experiments presented in Fig. 3(c) of the main paper are shown in Supplementary Figure 4(a) and (b), respectively. Similar to the data presented in Supplementary Figure 3 we made sure that no unwanted tip change occurred by performing another set of test measurement *after* scanning MnO_2/Ir(001). These data are presented in Supplementary Figure 4(c) and (d). The close agreement of the observed contrasts as well as the tunneling spectra measured at the locations indicated by red and blue dots in Supplementary Figure 4(b) and (d) indicate the absence of any tip change. This is also confirmed by a fit of domain wall profiles extracted from Supplementary Figure 4(b) and (d), which both result in the same tip magnetization angle with respect to the surface normal, i.e., $\theta \approx 80°$ [6].

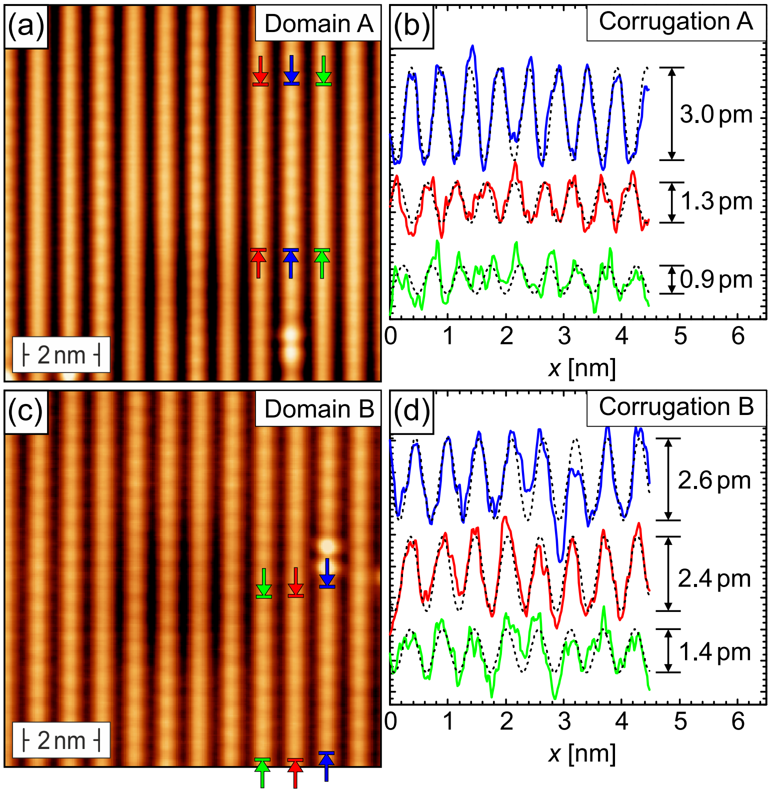

Supplementary Figure 5. SP-STM on two differently oriented MnO_2/Ir(001) domains, (a) domain A and (c) domain B. The respective line sections measured in between the colored arrow are shown in panels (b) and (d). These corrugation data, which have been obtained with the tip characterized in Supplementary Figure 4, were used in Fig. 3(c) of the main article. Stabilization parameters: $U = 1$ V, $I = 3$ nA.

(c) In-plane sensitive SP-STM on orthogonal MnO_2 chains on Ir(001)

We have performed SP-STM measurement on two domains, A and B, which each show MnO_2 chains on Ir(001) with identical structural periodicities but which are rotated by 90° (see inset of Fig. 3(c) in main paper). These data are presented in Supplementary Figure 5(a) and (c) together with line sections taken on three adjacent lines in panels (b) and (d), respectively. The magnetic corrugation of each line as extracted from a sinusoidal fit provides the data points in Fig. 3(c) of the main paper. In summary, the combined data of Figs. 3, 4, and 5 lead to the conclusion that the magnetic structure of MnO_2 chains on Ir(001) consists of in-plane antiferromagnetic chains. The inter-chain coupling mediated by the Ir(001) substrate exhibits a chiral 120° rotation of azimuthal spin orientation.

Supplementary Note 4: Identification of chain chirality

In order to identify the chirality of the observed spin spiral we first qualitatively evaluate the effect a left (LC) or right circular (RC) rotating spin spiral has on a SP-STM measurement. Four scenarios which are sketched in Supplementary Figure 6 have to be considered. We recall that the magnetic contrast of SP-STM scales with the cosine of the angle θ between the magnetization directions of tip and sample. Therefore, as shown in the upper row of Supplementary Figure 6, for the LC spiral we expect a periodic sequence with high-low-medium (HLM) contrast for a positive θ and HML for negative θ. By changing from LC to RC spirals we also switch the contrast sequence to HLM for negative and HML for positive θ (lower row). It is quite obvious that a stable tip polarization is necessary to avoid a change of the contrast sequence by the rotation of the tip polarization.

To verify the chirality we performed SP-STM on eight independent domains marked by numbered boxes in the overview scan of Supplementary Figure 7. In order to avoid any artifacts caused by a different orientation of the fast scan direction with respect to the stripe orientation, in this series we exclusively imaged domains with stripes oriented in the [110] directions and all domains were imaged with the same scan direction. As indicated by white letters L, M, H, we can again recognize stripes with low-medium-high sequence contrast which can also seen in the line sections below each scan. The contrast sequence of the domains 1 to 5 with a stable tip is LMH. Unfortunately, when zooming into domain 6 the tip changed causing an altered contrast ratio. Nevertheless, an LMH sequence is still observed. There are two possible scenarios to explain that. First, a sign change of angle

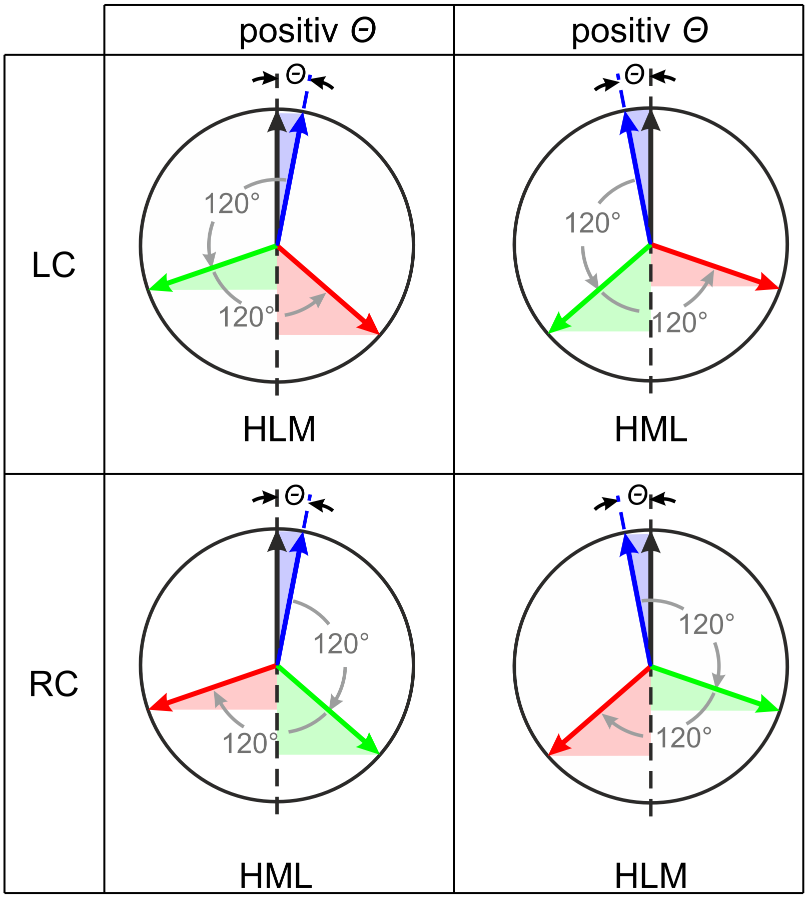

Supplementary Figure 6. Schematic representation to visualize the expected sequence of magnetic contrasts in dependence of the sample chirality (left-circular LR vs. right-circular RC) and the tilt θ between tip and sample magnetization. In case of an LC spin spiral a high-low-medium (HLM) sequence is expected for positive θ, a HML sequence for negative θ. Correspondingly, for a RC spin spiral the sequence is HML for positive and HLM for negative θ.

θ combined with an opposite rotational sense of the spin spiral or, second, a small change of the angle θ, which affects value but maintains the sign of θ. A similar tip change also takes place while scanning domain 7. At the position of the horizontal line a discontinuous change of the signal can be recognized in the line section. Again, this tip change changes the amplitude of the magnetic signal but keeps the LMH sequence in this and the following domain scan 8. Overall we have two series with a total of 7 independent SP-STM images. There is only a 2 % probability that this finding is accidental, strongly supporting that the spin spirals observed across the transition metal oxide (TMO) chains are indeed chiral.

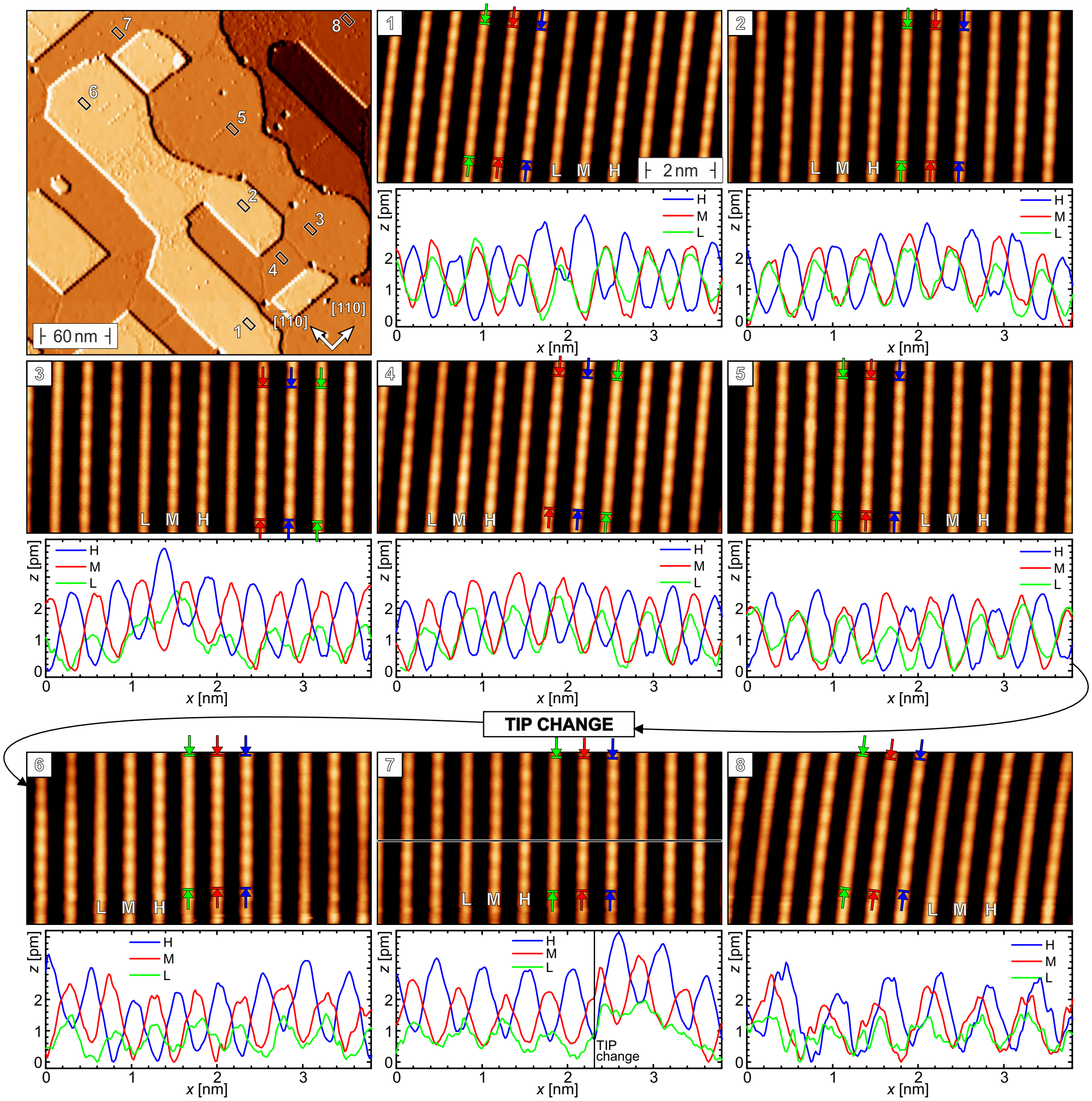

Supplementary Figure 7. SP-STM of eight independent domains labeled by boxes in the overview scan. Data sets 1–8 were scanned in the same direction on domains with the same stripe orientation. For scans 1–5 the tip was stable and a LMH contrast sequence was observed (cf. line sections below each scan). The tip change between data sets 5 and 6 affects the signal ratio of the contrast but not the sequence LMH in the scans 6–8. The very same effect is observed in domain 7 while scanning where a tip change is identified by jump of all of the three stripe signals. Stabilization parameters: $U = 300\,\mathrm{mV}$, $I = 300\,\mathrm{pA}$ for the overview scan and $U = 100\,\mathrm{mV}$, $I = 1\,\mathrm{nA}$ for data sets 1–8.

Supplementary Note 5: Theoretical analysis of the inter-chain spin interaction

At the first glance, the experimentally observed homochiral spin rotation from chain to chain looks similar to what was observed for thin Mn films on W(110) [10] where the Dzyaloshinskii-Moriya interaction (DMI) induced a spin-spiral structure with unique rotational sense. But a closer comparison shows that in the latter case the spins are spiraling in a plane formed by the surface normal and the propagation direction of the spin spiral, $\mathbf{q}$, so the rotational sense is determined by the Dzyaloshinskii vector $\mathbf{D}$ lying in the surface plane and perpendicular to $\mathbf{q}$. This mechanism is allowed by symmetry.

In the present case, the structure has a $(\bar{1}10)$ mirror plane (i.e. perpendicular to the MnO_2 chains) and according to Moriya's rules [11] this dictates the absence of a $\mathbf{D}$ vector (describing the interaction between the chains) in [110] or [001] direction. This is, naturally, also confirmed by our DFT calculations. On the other hand, to explain the experimentally observed homochirality a finite projection of $\mathbf{D}$ in the [001] direction is necessary. So we explicitly broke the $(\bar{1}10)$ mirror symmetry by displacing the Mn and O atoms with respect to each other in the chain direction and relaxing the structure again. After relaxation, the symmetry was restored, so that we can exclude this symmetry breaking as source for the observed spin structure. Note, that for all structural relaxations the generalized gradient approximation to the DFT exchange-correlation potential (like in Ref. [12]) was used.

As mentioned in the main text, a small oblique distortion of the (3×1) unit cell is observed in the STM images (also see the following Section VI of this Supplementary Material). Although this still preserves a two-fold rotational symmetry (with rotation axis along the surface normal) and thus excludes a z component of the $\mathbf{D}$ vector, it indicates that there might be details in the structure that are averaged out in a structural determination sampling over large surface areas and await further exploration. E.g. it can be expected that a finite MnO_2 chain shows structural relaxations at the ends that break the $(\bar{1}10)$ mirror symmetry

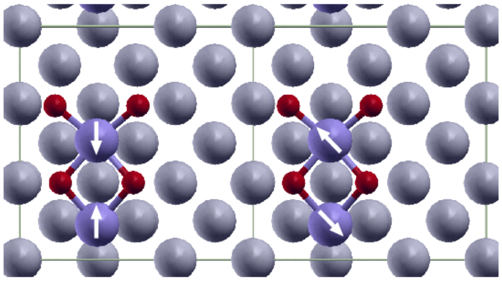

Supplementary Figure 8. Calculated structure of a short Mn_2O_4 chain on a (3×3) Ir(001) unit cell. The white arrows indicate the AFM coupling in the chain and the rotation of the Mn moments from chain to chain.

allowing for a finite D_z component. Whether such an edge effect leads to a significant change of the direction of the $\mathbf{D}$ vector depends on the ratio between in-plane potential gradient induced by the boundary and out-of-plane gradient induced by the surface. To estimate the size of the effects we conceived a rough structural model of finite MnO_2 chains which might possess an in-plane polar distortion. In a minimal model we relaxed a Mn_2O_4 chain on a (3×3) unit cell until the forces did not exceed 0.2 eV/a.u. (Supplementary Figure 8) and calculated the $\mathbf{D}$ vector components with an $8 \times 8 \times 1$ $\mathbf{k}$-point sampling. In this rough model it turns out that for $q = 0.33$ the ratio $D_z/D_y = 1.2$, meaning that $\mathbf{D}$ is about 50° tilted from the surface. The spins rotate in a plane that is 40 degree tilted from the surface showing that finite domains might give an effect that can be observed with in-plane tips. Such an effect at the edges of the domains could propagate into the domain itself by exchange interactions along the chains. Of course, a coupling mechanism between these structural distortions is needed to explain the homochirality of the domains. We speculate that dipolar interactions can play a role here and hope that further investigations of the surface geometry can clarify this issue.

Another possibility to account for the experimental observation is that the rotation plane favored by the DMI (i.e. the $(\bar{1}10)$ plane) is tilted by some other interaction around the [110] direction. E.g. dipolar interactions can favor spins rotating in the (001) plane, so that a spin-spiral can still be observed experimentally with an in-plane magnetized tip. But on the one hand, the dipolar interactions are very small due to the antiferromagnetic order within the chains, on the other hand our calculations indicate that such a tilt is also disfavoured by the magnetocrystalline anisotropy energy since it forces the spins to rotate over the medium $[\bar{1}10]$ axis while the easy axis is in [001] direction.

Finally, we add some details about possible errors arising in the DFT calculations. To account for correlation effects in the MnO_2 chains we applied the DFT+U method with values for the parameters U and J chosen to match the experimental data. Of course, there is no guarantee that a calculation that reproduces the experimental Mn d state dispersion also leads to the correct magnetic order of the Mn chains. In some systems, e.g. bulk Gd, such a correlation exists but the effects are also much bigger in that case [13]. Moreover, our calculations with different U parameters did not show significant changes in the results. Another complication that comes with the DFT+U calculations is that the magnetic force theorem, that simplifies the determination of the magnetocrystalline anisotropy and the

dispersion of the spin-spirals, cannot be used. Therefore, all calculations had to be performed self-consistently and this limits the cutoff parameters. For the shown results a planewave cutoff of 4.0 $(\text{a.u.})^{-1}$ and 864 **k**-points were used. For some cases the convergence was tested with higher values, but again no strong effects on the results were found.

Supplementary Note 6: Atomic scale STM

Supplementary Figure 9 shows two constant-current STM images measured with non-magnetic tips. In either case a striking left/right asymmetry can be recognized. In the case of Supplementary Figure 9(a) which was recorded on CoO_2 chains on Ir(001)at tunneling conditions of $U = 50\,\text{mV}$ and $I = 1\,\text{nA}$ the image is dominated by stripes along the $[\bar{1}10]$ direction (hatched black line). Along these stripes a weak corrugation can be recognized. As indicated by the continuous black line these maxima are not oriented perpendicular to the stripes but significantly rotated. We note that for the same material very similar images have been obtained in Ref. [12] (see Fig. 1c and Supplementary Figure S2 therein).

To our experience this result is not unique for CoO_2 but is also observed for other TMO chains on Ir(001). For example, the STM image presented in Supplementary Figure 9(b) was recorded on CrO_2/Ir(001) at $U = 200\,\text{mV}$ and $I = 0.5\,\text{nA}$. Again a stripe with a particularly high corrugation is marked by a hatched black line (though at a different position). Now we recognize a strong left/right asymmetry with a deep (dark) trench just right and a significantly shallower trench just left of this line. These result indicate that also the system

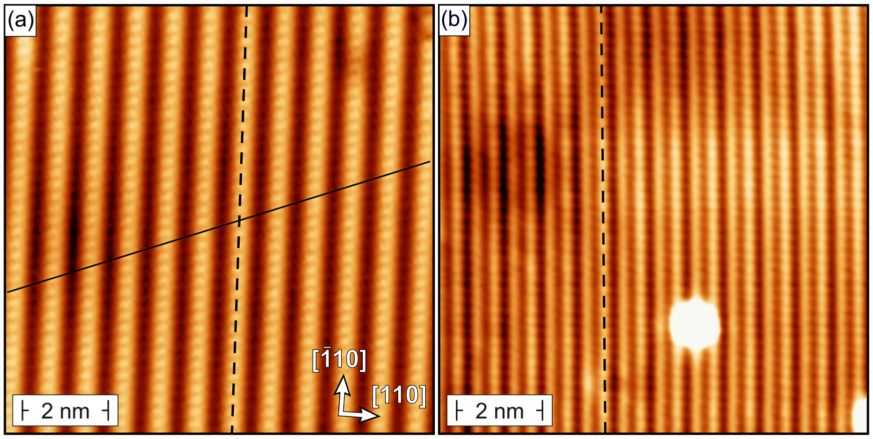

Supplementary Figure 9. Constant-current STM images obtained with non-magnetic probe tips on (a) CoO_2 and (b) CrO_2 chains on Ir(001). Stabilization parameters: (a) $U = 50\,\text{mV}$, $I = 1\,\text{nA}$ and (b) $U = 200\,\text{mV}$, $I = 0.5\,\text{nA}$.

of CrO_2 chains on Ir(001) is very complex and cannot be fully modeled by the simplified structure used for DFT calculations here and in Ref. [12].

* maschmitt@physik.uni-wuerzburg.de

[1] H. J. Elmers, J. Hauschild, and U. Gradmann, Phys. Rev. B **59**, 3688 (1999).

[2] O. Pietzsch, A. Kubetzka, M. Bode, and R. Wiesendanger, Phys. Rev. Lett. **84**, 5212 (2000).

[3] M. Bode, O. Pietzsch, A. Kubetzka, S. Heinze, and R. Wiesendanger, Phys. Rev. Lett. **86**, 2142 (2001).

[4] M. Bode, S. Heinze, A. Kubetzka, O. Pietzsch, X. Nie, G. Bihlmayer, S. Blügel, and R. Wiesendanger, Phys. Rev. Lett. **89**, 237205 (2002).

[5] O. Pietzsch, A. Kubetzka, M. Bode, and R. Wiesendanger, Science **292**, 2053 (2001).

[6] A. Kubetzka, O. Pietzsch, M. Bode, and R. Wiesendanger, Phys. Rev. B **67**, 020401 (2003).

[7] M. Bode, A. Kubetzka, S. Heinze, O. Pietzsch, R. Wiesendanger, M. Heide, X. Nie, G. Bihlmayer, and S. Blügel, Journal of Physics: Condensed Matter **15**, S679 (2003).

[8] E. Y. Vedmedenko, A. Kubetzka, K. von Bergmann, O. Pietzsch, M. Bode, J. Kirschner, H. P. Oepen, and R. Wiesendanger, Phys. Rev. Lett. **92**, 077207 (2004).

[9] S. Meckler, N. Mikuszeit, A. Preßler, E. Y. Vedmedenko, O. Pietzsch, and R. Wiesendanger, Phys. Rev. Lett. **103**, 157201 (2009).

[10] M. Bode, M. Heide, K. von Bergmann, P. Ferriani, S. Heinze, G. Bihlmayer, A. Kubetzka, O. Pietzsch, S. Blügel, and R. Wiesendanger, Nature **447**, 190 (2007).

[11] T. Moriya, Phys. Rev. **120**, 91 (1960).

[12] P. Ferstl, L. Hammer, C. Sobel, M. Gubo, K. Heinz, M. A. Schneider, F. Mittendorfer, and J. Redinger, Phys. Rev. Lett. **117**, 046101 (2016).

[13] P. Kurz, G. Bihlmayer, and S. Blügel, Journal of Physics: Condensed Matter **14**, 6353 (2002).
